# Dynamic capabilities, value creation and value capture: Evidence from SMEs under Covid-19 lockdown in Poland

**DOI:** 10.1371/journal.pone.0252423

**Published:** 2021-06-15

**Authors:** Wojciech Dyduch, Paweł Chudziński, Szymon Cyfert, Maciej Zastempowski

**Affiliations:** 1 College of Management, University of Economics in Katowice, Katowice, Poland; 2 Poznań University of Economics and Business, Poznan, Poland; 3 Institute of Management, Poznań University of Economics and Business, Poznan, Poland; 4 Department of Enterprise Management, Nicolaus Copernicus University, Torun, Poland; The Bucharest University of Economic Studies, ROMANIA

## Abstract

Dynamic capabilities, resulting from activities that allow conscious and skillful modification of a firm’s strategic potential, are seen as one of the key drivers of a firm’s value creation, competitive advantage and above-average performance in changing environments. However, little is known about how dynamic capabilities can shape business survival and performance during crises. The research objective of this paper is twofold. First, through a literature review, we seek to identify which first-order dynamic capabilities–managerial decisions under uncertainty—are vital for rapid response to a crisis. Second, we present the results of research carried out among 151 small and medium-sized companies in Poland immediately after the beginning of the economic lockdown (April 2020). The survey that we developed identifies which dynamic capabilities were essential for businesses to survive during this unexpected black swan event. We also present dependence and regression analyses showing the links between the identified dynamic capabilities and value creation, understood as retaining employees and production levels, as well as value capture, understood as maintaining cash flow and current revenues.

## 1. Introduction

The central task of contemporary strategic management is to look for sources of value and to achieve above-average firm performance. Moreover, strategy is defined as the dynamics of relations between an organization and its environment [1], in which resources and actions are committed in order to reach a sustainable competitive advantage [2]. To answer the question why certain organizations create more value than other, and why some of them achieve higher performance, strategic management has adapted various theoretical perspectives over time, the dynamic capabilities perspective being one of them.

The dynamic capability perspective focuses attention towards conscious and skillful modification of a firm’s strategic potential through strategic change aimed at achieving above-average performance. A recently conducted meta-analysis demonstrates that dynamic capabilities translate into higher overall firm performance, specific outcomes either in the domain or processes, external environment-organization fit, enterprise survival, growth, flexibility reflecting the ability to accommodate major changes, and innovativeness outcomes such as new products, patents, resource portfolio changes, organizational learning, etc. [3].

Dynamic capabilities can be analyzed as an organization’s overall portfolio of capabilities operating on two levels [4]. Operational capabilities (the first level) include routine activities that allow organizations to pursue a defined set of activities. Above operational capabilities are dynamic capabilities, including first-order and higher-order capabilities (second level [5]). First-order dynamic capabilities include managerial decisions taken during uncertainty, while high-order dynamic capabilities facilitate the sensing and seizing of new or changed opportunities [6]. Dynamic capabilities, which influence the pace of an organization’s adjustment processes, enable companies to create and capture value, thus helping them to survive during rapid changes in the environment.

The dynamics of changes in the environment following the coronavirus crisis, which has led to the shutdown of global economies, encouraged us to undertake research on the impact of dynamic capabilities on the ability of SMEs to create and capture value in crisis situations, shedding new light on the nature of dynamic capabilities. By focusing on assessing how dynamic capabilities exert an impact on companies in a crisis, we draw certain conclusions as to which capabilities influence the value creation and capture processes.

The challenge of value creation is typically analyzed together with the innovation capability of organizations. Indeed, new or changed opportunities that are discovered or generated, when diligently prepared as innovations, can be an important source of value creation [7]. A recent study shows that proper strategic orientation, organizational innovation capability and strategic planning influence company performance [8]. However, even the most valuable and useful innovations will not translate into performance if innovative organizations are not able to capture a proportion of the value.

Some firms are able to capture more value than others, even though they do not create it or create a small proportion of value. This proves that possessing or controlling resources is not the only necessary condition for creating value. What is also required is the ability to use resources to generate higher value and to capture such value [9]. This problem is connected with dynamic capabilities and the managerial competences to bundle, transform and orchestrate resources in order to quickly and flexibly respond to opportunities that emerge in the environment [2]. Our research on the impact of dynamic capabilities on value creation and capture during the Covid-19 crisis sheds some light on the survival of businesses during turbulent times, and the role of dynamic capabilities in the process of sustaining performance.

In the first part of the paper, we use literature analysis to identify the specific dynamic capabilities (first-order dynamic capabilities) that are connected with bundling, transforming and orchestrating resources to quickly respond to unexpected changes in the environment. In the next section, we determine the significant indicators of value creation during crises: retaining employees and maintaining production levels (thus securing the market share). We also discuss value capture elements, that is maintaining both cash flow and revenue streams. In the next section, we present the results of our CAWI-based research carried out among 151 micro, small and medium-sized companies a month after the March 2020 lockdown in Poland. The results indicate how specific organizational capabilities and competences, which shape companies’ higher-order dynamic capabilities, influence the value creation and value capture processes.

## 2. Theoretical underpinnings and conceptual framework

There is a relatively rich body of literature related to dynamic capabilities, value creation and value capture, as well as to managerial responses during economic crises, particularly in the context of the most recent financial crisis of 2007–2009, or the SARS epidemic in 2003. However, there is still a gap concerning the relations between dynamic capabilities and value creation and capture during turbulent times. The following section describes the construct of dynamic capabilities, identifies the most frequent first-order dynamic capabilities developed during crises, and presents the value creation and capture processes. We sought to identify the most crucial organizational abilities during times of crisis, and find both the relations between them and various measurements for value creation and capture.

### 2.1 Dynamic capabilities

Dynamic capabilities, understood as reconfiguring a company’s resource base in order to better sense and seize opportunities, are seen as one of the key drivers of a firm’s performance in changing environments. They focus managerial attention on conscious and skillful modification of the firm’s strategic potential [3].

Dynamic capabilities constitute a set of capabilities that operate on three levels [4]. At zero level, there are ordinary capabilities, known as substantive or operational capabilities, embrace routine activities that allow organizations to pursue a defined set of activities. Above these are dynamic capabilities, which include first-order and higher-order capabilities [10]. First-order dynamic capabilities concern managerial decisions taken during uncertainty (the topic of our paper), while higher-order capabilities facilitate the sensing and seizing of new or changed opportunities [6]. While it is higher-order capabilities that managers should focus on since they shape business strategies, influence the processes of sensing and seizing opportunities and address the problems that organizations wish to solve [11], it is also important to identify which first-order dynamic capabilities are the most crucial for value creation during times of crisis, and which of them constitute higher-order dynamic capabilities. The focus of this paper is to identify the most likely managerial responses during crises, seen as first-order dynamic capabilities [4].

Dynamic capabilities, which influence the pace of an organization’s adjustment processes, enable companies to survive during rapid changes in the environment. It is worth noting that dynamic capabilities play a unique role in the functioning of SMEs, which—due to the limited resources they have or have access to—are more sensitive to crises than large companies. The literature indicates that dynamic capabilities make it possible to maintain a competitive position in changing environments in the long term. At the same time, there is little research that would refer to their impact on decision-making processes in the short term. The dynamics of changes in the environment following the coronavirus crisis, which has led to the shutdown of global economies, encouraged us to undertake research on the impact of dynamic capabilities on the ability of SMEs to create and capture value in crisis situations, shedding some new light on the nature of dynamic capabilities and their lower-level structure. By focusing on assessing how dynamic capabilities exert an impact on companies in a crisis, we draw some conclusions as to which first-order capabilities influence the value creation and capture processes.

Dynamic capabilities, which are also seen as competences in the field of detection, acquisition and transformation [12], constitute a set of processes that ensure an organization is able to cope with changes in the competitive environment [13], and thus influence the management’s ability to manage strategic changes [14]. G. Zhang *et al*. [15] indicate that by influencing schemes and procedures, dynamic capabilities ensure adjustment of an organization’s architecture, while operational capabilities are related to activities ensuring the efficiency and stability of an organization’s functioning. This approach, highlighting the link between activities, resources and dynamic capabilities [13], indicates a high level of complexity of dynamic capabilities [16].

Dynamic capabilities influence a firm’s performance. It turns out that environmental dynamism negatively influences the contribution of ordinary capabilities and positively influences the contribution of dynamic capabilities to a firm’s performance [17]. What is more, heterogeneity strengthens the contribution of dynamic capabilities to a firm’s performance.

Recent results indicate that supply chain dynamic capabilities positively influence technological innovation and operational performance of firms [18]. It is worth noting that dynamic capabilities also have an influence on firms’ performance only when mediated by marketing ability [19]. At the same time, not all performance measures can detect the influence of dynamic capabilities. This points to the importance of social capital in acquiring and transforming resources as the essence of dynamic capabilities, but also in capturing value [20].

Some first-order dynamic capabilities such as marketing skills, including identification of market specifics, brand management and customer service, have a direct impact on increases in an organization’s profits [21]. Dynamic capabilities are also the essence of modern competitive strategies [22]. Understood as the ability to sense weak signals from customers and make strategic choices on this basis, they have a significant impact on the competitiveness of organizations [23].

In the discussion on dynamic capabilities, three assumptions need to be made. Firstly, we posit that it is important to delineate between operational or substantive capabilities and dynamic capabilities (second order and higher order), keeping in mind that the latter indirectly shape organizational performance by changing substantive capabilities. This paper does not analyze substantive capabilities, only first-order dynamic capabilities. Secondly, we assume that dynamic capabilities are a strategic variable that have to be analyzed on the organizational level. Thirdly, we assume that dynamic capabilities are a multidimensional construct, which avoids arguments based on each organization’s specificity or dynamic capability universality. Our aim is to demonstrate which first-order dynamic capabilities are important during times of crisis and how they can shape value creation and capture, thus becoming higher-order dynamic capabilities that shape a firm’s performance.

### 2.2 Dynamic capabilities during turbulent times

Research on dynamic capabilities sheds some light on the survival of businesses during turbulent times, and indicates the role of dynamic capabilities in the process of sustaining performance. The literature demonstrates some evidence based on managerial responses during times of crisis. Battisti and Deakins, for example [24], when examining the dynamic capabilities of SMEs after the 2010–2011 earthquake disaster in Christchurch, New Zealand, indicate the importance of dynamic capabilities expressed in a proactive attitude and the ability to integrate resources when identifying new opportunities in an environment characterized by high volatility and increased uncertainty.

The Covid-19 pandemic, which emerged at a time when the world economy is more interconnected than ever [25], represents an unprecedented external shock [26] forcing the rethinking of business models [27]. Liguori & Pittz suggest that the economic fallout from this pandemic will prove worse for small businesses and their employees before it gets better [28]. Syriopoulos suggests that many SMEs are expected to go under during and after the Covid -19 crisis [29]. The restrictions imposed to prevent the spread of the Covid-19 outbreak have had more severe effects on SMEs than on larger and global firms because they have lower capital reserves, fewer assets and lower levels of productivity [30]. Similar conclusions regarding the resilience of SMEs are formulated by Lu et al., who examined the impact of the Covid-19 epidemic in Sichuan Province and suggested that SMEs are often most vulnerable when there are major public crises due to their lower levels of preparedness [31]. The negative impact of the Covid-19 crisis in Russia is indicated in research by Razumovskaia et al., which suggests that the vulnerability of this sector of the economy consists not only of limited resources, but is also due to a relatively low level of innovation potential [32]. Studying the impact of the Covid-19 pandemic on Spanish companies, Pedauga et al. note that while the maintaining of economic activity shows greater sensitivity to the behavior of large firms, employment depends substantially on SMEs in general and microenterprises in particular [33]. Gourinchas et al., who conducted research on the impact of the Covid-19 crisis on business failures among SMEs in seventeen countries, estimate a large increase in the failure rate of SMEs during Covid-19 [34].

Scholars distinguish five categories of substantive capabilities and six categories of higher-order dynamic capabilities [35]. Substantive capabilities embrace those activities that are essential for an organization’s survival in the short term: (1) operations/processes, (2) product/service/quality, (3) resources/assets, (4) organizational structure, and (5) relationships with customers/buyers. In turn, dynamic capabilities relate to an organization’s activities which form the basis for its long-term development, namely: (1) R&D/innovation/technology, (2) strategic decision-making/market research, (3) cooperation/alliances/networks/relations, (4) knowledge management, (5) intangible resources/reputation, and (6) strategic human resource management.

It would be challenging to study higher-order dynamic capabilities without knowing their structure and composition. To study dynamic capabilities it is important to identify which first-order dynamic capabilities make up this construct in turbulent times. As a consequence of a literature review, we have identified the following first-order dynamic capabilities that are seen as the most crucial in the context of the coronavirus crisis (Table 1): (a) The ability to obtain financing; (b) The ability to work in virtual teams; (c) Delegation of power and greater autonomy of employees; (d) The ability to take advantage of opportunities that appear during a crisis; (e) The ability to innovate and/or imitate; (f) The ability to differentiate between products and services offered; (g) The ability to use and develop modern technologies; (h) The ability to move resources quickly; (i) Good work organization and proper planning; (j) The ability to maintain and develop efficient IT systems; and (k) The ability to use personal relationships. Below, we develop the theoretical underpinnings behind each of these capabilities.

**Table 1:** First-order dynamic capabilities in turbulent times.

Dynamic capabilities	Authors
The ability to obtain financing	Sameen & Cowling, 2015 [36]; Arrfelt *et al*. 2015 [37]; Bigler & Hsieh, 2016 [38]; Sammut *et al*., 2017 [39]; Townsend & Busenitz, 2015 [40]
The ability to work in virtual teams	Monalisa *et al*., 2008 [41]; Wadsworth & Blanchard, 2015 [42]; Mukherjee *et al*. [43], 2012; Brahm and Kunze, 2012 [44].
Delegation of power and greater autonomy of employees	Dirani et al., 2020 [50]; Felin & Powell [46], 2016; Klein et al., 2006 [49]; Sommer et al., 2016 [48]; Uhlaner et al., 2013 [47]; Wohlgemuth et al., 2019 [45]
The ability to take advantage of opportunities that appear during a crisis	Oliver and Holzinger, 2008 [52]; Harreid *et al*., 2007 [53]; Teece, 2016 [54]; Marsh and Stock, 2003 [55]
The ability to innovate and/or imitate	Nassimbeni, 2001 [57]; Lawson & Samson, 2001 [64]; Romijn & Albaladejo, 2002 [65]; Akman & Yilmaz, 2008 [60]; Martinez-Roman *et al*., 2011 [56]; Dziallas & Blind, 2019 [61]; Zhang & Merchant, 2020 [66]; Mendoza-Silva, 2020
The ability to differentiate between products and services offered	Calantone *et all*., 2002 [72]; Subramaniam & Youndt, 2005 [73]; Cheng & Yang, 2017 [74]; Dziallas & Blind, 2019 [61]
The ability to use and develop modern technologies	Leoncini *et al*., 2019 [75]; Zagelmeyer & Heckman, 2013 [76]; Kalman & Hernandez, 2018 [77]; Mortazavi *et al*., 2020 [78]; Dalić & Paunović, 2017 [79]
The ability to move resources quickly	March, 2014, Zhang 2007 [84], Teece, 2007 [5]; Karim & Capron, 2015; Maritan 2001 [81]; Hitt, Ireland, Sirmon & Trahms, 2011; Wilden, Devinney and Dowling 2016 [83].
Good work organization and proper planning	Desai et al., 2007 [90]; Felin & Powell, 2016 [46]; Ghapanchi & Aurum, 2012 [89]; Holzweber et al., 2012 [91]; Messersmith & Guthrie, 2010 [88]; Ojha et al., 2020 [85]; Popadiuk et al., 2018 [87]; Schwarz et al., 2020 [86]
The ability to maintain and develop efficient IT systems	Desai et al., 2007 [90]; Guo et al., 2020 [94]; Khatri et al., 2010 [97]; Kim et al., 2011 [96]; Wamba et al., 2020 [92]; Wang et al., 2013 [93]; Yoshikuni & Albertin, 2017 [95]
The ability to use personal relationships	Fath et al., 2021 [101]; Portuguez Castro & Gómez Zermeño, 2020 [102]; Mitręga, 2017 [99]; Sachitra & Chong, 2018 [98]; Salvato & Vassolo, 2018 [100]

#### 2.2.1. The ability to obtain financing

In times of crisis, a typical concern is that access to obtaining finance is an increasingly significant barrier to business growth [36]. Arrfelt *et al*. [37], who assume that competences in the field of capital allocation are a form of dynamic capabilities, indicate the relationship that exists between the level of competences in the field of allocation and the company’s development prospects, as well as the importance of competences in the field of allocation in competitive markets. Research by W. Bigler and Ch. Hsieh [38] also indicates the importance of dynamic capabilities in the process of optimizing the capital structure, according to which the use of the construct of dynamic capabilities allows a company’s assets to be remodeled in such a way as to ensure the optimization of both profits and company value. Similarly, Sammut *et al*., [39], in their study into the ability to develop academic spin-offs (ASOs) in France, indicate four factors that determine the growth of ASOs: entrepreneurial orientation, acquiring skills in the entrepreneurial process, access to public and private financial resources, and technological opportunities and support programs. D. Townsend and L. Busenitz [40] use the construct of dynamic capabilities to describe the extent to which, at the initial stage of a company’s development, the choices made between the quality of the management team, the company’s technological resources and the uncertainty of demand in key markets affect the ability of companies to acquire financial resources.

#### 2.2.2. The ability to work in virtual teams

Building virtual teams is a significant challenge for managers during pandemics, lockdowns or remote working. The current crisis has forced companies to quickly invest in online technologies and reorganize their teams into virtual ones. To date, research indicates that smaller, tightly-knit teams have higher success rates [41], but there is also a need for better communication between members of virtual teams in terms of goals and tasks, in addition to the social and emotional aspects. Being part of teams that work remotely is a challenge for leaders, whose existing tactics of exerting influence during in-company work may be insufficient [42] Meanwhile, the understanding and recognition by leaders of team members’ specific set of competencies at a given stage of a team’s development, result in increased team effectiveness and have a positive effect on the social environment [43]. Brahm and Kunze [44] point to the importance of group trust as a critical factor in achieving high performance among virtual teams.

#### 2.2.3. Delegation of power and greater autonomy of employees

Economic crises and times of uncertainty require quick responses from organizations to prepare innovative solutions and create new value for customers. These responses cannot happen in hierarchical, concentrated and centralized structures. In order to respond quickly to an unexpected crisis, companies need to develop organic structures with greater autonomy, power decentralization, creative idea generation and bottom-up experimenting [7]. Wohlgemuth *et al*., [45] argue that in research on dynamic capabilities, attention is usually focused on the behaviors of managers identifying and implementing business opportunities. However, the role of employee involvement and participation is usually ignored, which, as their research shows, positively correlates with shaping dynamic capabilities. Felin and Powell [46] point to new organizational forms such as polyarchy, social evidence and new forms of open organization, which, through employee involvement, enable organizations to build dynamic capabilities in the area of sustainable innovation. The positive impact of employee involvement on process innovations is also emphasized by Uhlaner *et al*., [47] who study factors affecting sales growth in SMEs related to knowledge and innovation.

Sommer et al. explore the influence of leader behavior on team members’ resilience, and indicate that delegation, connected with transformational leadership, was associated with greater levels of positive effects and lower levels of negative effects, which in turn predicted higher resilience among team members [48]. Similar observations were made by Klein et al., who suggested that dynamic delegation enhances an extreme action team’s ability to perform reliably, while also building their novice team members’ skills [49]. Dirani et al. point to the fact that the role of top leadership is to utilize team delegation in order to come up with efficient roadmaps for achieving goals and responding to crises. Delegation during a crisis situation strengthens teams, improves decision-making, and boosts stakeholders’ commitment to the organization and its survival [50].

#### 2.2.4. The ability to take advantage of opportunities that appear during a crisis

What may seem a threat to one organization can be an opportunity for another. Crises typically put organizations in financial dire straits, and some of them experience difficult times. The role of dynamic capabilities is to react to crisis situations, sense new opportunities and reconfigure the company base and resources in order to seize any opportunities sensed. Opportunity exploitation during a crisis is understood as the sum of new products and services launched, or new markets penetrated [51]. The literature has already identified the links between dynamic capabilities and the effectiveness of implemented strategies [52]. The disciplined implementation of strategies as well as the dynamic transformation of some of them show that companies are able to sense changes in the market and take opportunities by reconfiguring their resources and competences, which is an expression of the development of their dynamic capabilities [53].

Dynamic capabilities favor entrepreneurial management based on sensing opportunities, developing applicable business models and stimulating innovativeness [54]. Dynamic capabilities are also an important condition for the innovativeness of companies and the development of new products. It turns out that they also stimulate the development of technological and marketing skills, which can later be used for the development and commercialization of new products [55].

#### 2.2.5. The ability to innovate and/or imitate

The CEO of one of the companies that we surveyed, when asked if he would save costs on R&D and innovations due to the crisis said “Never–we want to develop new products right now so that we become stronger after the crisis”. The ability to innovate seems to be crucial even during times of crisis. The ability to innovate and/or imitate is usually identified with the innovation capability [56,57], which stems from dynamic capabilities [58,59]. The ability to innovate itself is perceived and understood in various ways [60–63]. Lawson and Samson suggest that innovation capability is the ability to continuously transform knowledge and ideas into new products, processes and systems for the benefit of the firm and its stakeholders [64]. Based on dynamic capability theory, Romijn and Albaladejo understood innovation capability to be the skills and knowledge needed to effectively absorb, master and improve existing technologies, and to create new ones [65]. Zhang and Merchant indicate that it is the ability to create better or more effective products, processes, services, technologies or ideas that are accepted by markets, governments and society [66]. However, Wang believes that dynamic innovation capabilities are innovation capabilities that firms use to develop, integrate and reconfigure resources and operational capabilities [67]. On the other hand, Mazzucchelli *et al*. suggest that there are strategic innovation capabilities that can be considered as the ability to develop new products, implement innovations in the manufacturing process, carry out product improvements, and apply innovations in marketing and methods [68]. It is also worth emphasizing that the innovation capability is indicated as one of the key areas that determines the innovativeness of SMEs [56,69–71].

#### 2.2.6. The ability to differentiate between products and services offered

An essential second-order dynamic capability that shapes overall dynamic capabilities during turbulent times is the ability to differentiate between the products and services offered, which is often treated as an element of the innovation capability. Calantone *et al*. indicate that the determinants of a company’s innovation include its ability to use new ideas or search for new ways of doing things [72]. On the other hand, however, Subramaniam and Youndt suggest that the key element in implementing minor innovations is a company’s ability to reinforce prevailing product/service lines and strengthen existing expertise in prevailing products/services [73]. Akman and Yilmaz indicate that for innovation, it is important how quickly the company is able to adapt its offer to changing market conditions [60]. Similarly, C. Cheng and Yang focus on the role of this ability in developing new products to satisfy market needs [74].

#### 2.2.7. The ability to use and develop modern technologies

As has already been mentioned, the coronavirus crisis has forced companies to work online. Even if firms had been developing their IT systems before, the lockdown speeded the process up. In normal times, technological development contributes to the growth of companies, including those using ‘green technologies’ [75], which has a positive effect on their ability to survive a crisis. Zagelmeyer and Heckman [76] indicate that both the size of the company and the business problems occurring before a crisis have a positive effect on the ability to survive the crisis, while a flexible employment structure (temporary or fixed-term employees) did not affect the company’s survival during the crisis. The development of technology, especially know-how in the field of information technology, has a positive impact on a company’s survival and its adaptation to the changing requirements of the environment, also in the case of micro-companies [77]. Mortazavi *et al*. [78] point to the legitimacy of using artificial intelligence to provide a higher level of security in the fight against the Covid-19 coronavirus by analyzing which employees are at highest risk of contracting the virus. What is more, artificial intelligence can also support areas outside business such as education, healthcare or public services in the fight against the coronavirus. According to the results of research carried out by Dalić and Paunović [79], more and more SMEs are using information technologies to achieve better and better results, and those that use IT technologies are more resistant to external disturbances and are more able to survive.

#### 2.2.8. The ability to move resources quickly

The essence of dynamic capabilities is the ability to spot opportunities, reconfigure the resource base in order to seize them, identify new opportunities, and transform the whole company for the future (Teece, 2007) [5]. Especially during unexpected crises, instead of sinking into despair, companies should turn the situation to their benefit as much as possible. In extreme cases, this can be done by changing the type of production, entering new markets or diversifying. However, no matter which course of action is chosen, when a new opportunity is spotted, it requires resources to be quickly shifted and strategic potential to be directed towards seizing the opportunity.

Moving resources quickly, or resource reconfiguration, is understood as the dynamic capability which embraces fitting resources to new decisions [80]. Maritan [81] explains shifting resources through such behaviors as seeking resources, and their selection, investment, exploitation and reconfiguration. Hitt, Ireland, Sirmon and Trahms [82], on the other hand, understand resource orchestration as a key element of strategic entrepreneurship. According to Wilden, Devinney and Dowling [83], resource reconfiguration concerns investing in new businesses, deploying existing businesses, alliance creation or business model adaptation. The dynamic capability, understood as making rapid responses to signals coming from the environment and shifting resources quickly, translates into the effectiveness of an organization in terms of its profitability and work productivity, especially if it is accompanied by an efficient IT system [84].

#### 2.2.9. Optimal work organization and proper planning

Karna et al. [35] distinguish some dynamic capabilities, among which they mention proper organizational structure and human resource management. D. Ojha, P. Patel and S. Sridharan [85] indicate that although dynamic strategic planning is negatively or insignificantly related to financial results, it also has a positive impact on financial results through operational capabilities. J. Schwarz, R. Rohrbeck and B. Wach [86], while emphasizing the role of leadership training and building corporate forecasting practices in shaping dynamic capabilities, also value the mediating role of dynamic management opportunities (*i*.*e*., the ability of leaders to question current business models, make decisions during uncertainty, and reconfigure organizational resources). Research conducted by S. Popadiuk, A. Luz and C. Kretschmer [87] points to the relationship between the concept of dynamic capabilities and ambidexterity, and emphasizes the importance of organizational learning, sources of information, organizational projects, management of limited resources, resource growth through outsourcing and alliances, the role of top management and team participation in decision-making, and the importance of asset specialization and organizational management. When examining the determinants of success and failure of developing companies, Messersmith and Guthrie [88] indicate that high-performance work systems are positively related to sales growth and innovation through the prism of the dynamic capabilities concept. Ghapanchi and Aurum [89] show how dynamic capabilities related to proper work organization—that is proactive and effective defect removal, as well as proactive and efficient functionality enhancement—influence the higher efficiency of Open Source Software projects. Desai *et al*. [90] indicate that the sources of competitive advantage for the CRM process in terms of dynamic capability are the ability of an organization to continuously improve, innovate and reconfigure resources to adapt to changing environmental needs. Furthermore, studies by Holzweber *et al*. [91] indicate that the development of dynamic capabilities, determined by the actions of management and key clients (in particular in the area of information exchange and coordination), influences the improvement of business processes and the establishment of a strategic partnership with the client’s organization.

It also seems that the right organization design has an impact on the development of dynamic capabilities. In a changing environment, it is the openness of an organization, loose organic structures and self-management processes that are important [46].

#### 2.2.10. The ability to maintain and develop efficient IT systems

Efficient IT systems become crucial for company activities during crises when remote working, cloud storage and connection-intensive online sessions are required. Research by Wamba *et al*. [92] shows the impact of dynamic capabilities based on big data analysis (BDA) on the improvement of organizational efficiency and organizational performance, supply chain agility (SCAG), supply chain adaptability (SCAD) and performance measures (cost and operational efficiency). Wang *et al*. [93] adopted the assumption of dynamic capabilities as an intermediary variable, and presented results which indicated that IT support indirectly benefits production organizations, which forces companies to carefully adjust IT support to strategic needs. Also, Guo et al. argue that digitalization has the potential to help SMEs respond effectively to public crises by activating their dynamic capabilities [94]. Moreover, studies by D. Desai, S. Sahu and P. Sinha [90] indicate that competences in the field of IT are a significant moderator of the dependence between dynamic capabilities and competitive advantage. A similar conclusion is formulated by Wang *et al*. [93], who indicate a significant direct impact on dynamic marketing opportunities of a company’s market orientation, the use of IT to support CRM and the functionality of IT infrastructure.

Yoshikuni and Albertin [95] indicate that dynamic operational and analytical capabilities related to IT services have a positive impact on improving business processes and company performance. By examining dynamic process-oriented capabilities, Kim *et al*. [96] indicate the strategic role of IT as a determinant of dynamic capabilities in improving a company’s performance. At the same time, research by Khatri *et al*. [97] indicates the advisability in dynamic capability studies of focusing on HR and IT capabilities rather than HR practices or IT investments as sources of sustainable competitive advantage, since the capability construct fits more closely to the definition of ‘resource’ than HR practices or IT investments.

#### 2.2.11. The ability to use personal relationships

Bilton and Cummings [7] suggest that during turbulent times, leadership based on building relations inside and outside organizations is as important as leadership based on building a strong vision. Studies carried out by Sachitra and Chong [98] on small Sri Lankan companies in the agribusiness sector provide evidence of a link between a competitive advantage and dynamic capabilities such as organizational learning, relationship building, quality management and marketing. However, research by Mitrega [99] indicates the possibility of using an organization’s dynamic capabilities in the process of creating interpersonal skills.

Salvato and Vassolo [100], in trying to explain the dynamics of resources, proposed a multi-level theory of dynamic capabilities (DC) which indicates the central role of the ability of individual employees to use interpersonal relationships conducive to a productive dialogue.

Also, Fath et al. examined how relationships have influenced SME resilience during the Covid-19 crisis, and suggest that strong ties increase resilience even when there is a negative outlook, as network partners support each other, including through the development of new ties [101]. Portuguez Castro & Gómez Zermeño draw similar conclusions, indicating that social and human capital have increased resilience during the Covid-19 crisis [102].

### 2.3 Value creation and value capture

Studies demonstrate that shaping and developing dynamic capabilities translates into value creation and capture that can be treated as the outcome of dynamic capabilities [103]. Most of the value is created at the beginning of the product or service development process, during creative idea generation or discovery. Therefore, it is vital for organizations to secure the processes that result in value creation: recruiting and maintaining creative staff to develop innovations, as well as securing complementary resources and production processes to meet market demand. Organizations need to take care of various value-related processes: adding value—understood as incremental continuous improvement of existing value; creating value—understood as new product, service or technology preparation; as well as co-creating value—a combination of both that becomes a natural strategic choice and a dominant logic in marketing [104].

Contemporary organizations create value by combining the resources they control with resources owned or controlled by customers, suppliers and end users [105]. Value creation makes it possible for organizations to identify consumers’ needs and preferences, which are harder to analyze from a classical perspective [106]. Ultimately, organizations may wish to create an innovative ecosystem, which is seen as a driver of creating and capturing value [107].

Value is explained as a dimension of a firm’s performance [50]. If performance is treated as a multi-dimensional construct that embraces long-term financial performance (from a customer, shareholder or managerial perspective in relation to competitors) and short-term returns in stock, then value is the proportion of financial performance that occurs to relevant stakeholders. In this paper, we see financial liquidity and the revenue stream as important indicators of a firm’s ability to capture value. In order to sustain value creation and capture at a satisfactory level, organizations seek to realize goals that will drive performance and growth [108]. Of course, the goals realized during regular functioning of an organization will differ from the goals set during times of crisis.

Previous studies on the ability of companies to survive in crisis conditions indicate the importance of measures taken to retain employees–in particular skilled employees who can create value from the moment of idea generation to innovation commercialization [109,110] and maintain financial liquidity as a condition for the further functioning of a company [111]—making them desirable elements of value creation and value capture.

#### 2.3.1. Retention of existing employees for value creation

Dynamic capabilities can be important in hiring employees and managing human resources [112]. During crises, shortages of resources for the implementation of an organization’s tasks are particularly noticeable, and therefore, the resources owned should be used in the most effective way. This applies in particular to human resources, their competences and skills, but also to the feeling of providing employees with a sense of security, and appreciating employees by delegating tasks and ensuring a sense of financial stability, which is particularly difficult in the event of a financial crisis [109]. Retaining essential employees is a challenge for human resources management, especially in times of crisis, when some employees are ready to return to work from retirement or delay their retirement. SMEs are more vulnerable to the crisis, and therefore, react more dynamically when compared to large companies by reducing the level of employment during a crisis [113]. Retaining employees, and skilled employees in particular, is a prerequisite for value creation based on new and useful ideas, preparing innovations and launching them onto the market.

#### 2.3.2. Maintaining current production for value creation

Another prerequisite of value creation during crises is being able to meet the customer demand for products that offer unique value. For companies, it is not enough to be creative and innovative—innovations need to be launched onto the market and readily available. Although crises have a negative impact on the level of production, in some countries, export-related companies are able to quickly rebuild their production capacity and restore supply chains damaged by a crisis [114]. The decisions made by managers of companies during a pandemic are aimed at securing the health of employees and clients, and thus maintaining the production capacity of their companies [115].

Experience to date with the impact of epidemics on agricultural production has shown a significant decline in production in regions most affected by the Ebola virus epidemic in Liberia [116].

#### 2.3.3. Maintaining financial liquidity as a value capture mechanism

In the next section, two elements will be discussed: maintaining financial liquidity and current revenues, as these are outcomes that define value capture by organizations. At first glance, the dependence between dynamic capabilities and the flow of money may seem controversial. After all, the shaping and development of dynamic capabilities take place over a long period and are of a strategic nature, while maintaining the flow of money is an operational activity [117]. However, in the literature, certain links have been identified between dynamic capabilities and effectiveness understood as maintaining money flow over a longer period [111], primarily through the prism of implemented strategies [52]. The disciplined implementation of strategies, as well as the dynamic transformation of some of them show that companies are able to sense changes in the market and take opportunities by reconfiguring their resources and competences, which is an expression of the development of their dynamic capabilities and leads to a stable financial situation [53]. Unique dynamic capabilities, such as technological and marketing abilities, translate into maintaining money flow and efficiency [21]. However, it is very difficult to determine which abilities and reconfigured resources translate into money flow growth; as usual, it is a combination, which also includes the strategic responses of competitors [5].

Dynamic capabilities favor entrepreneurial management based on sensing opportunities, developing applicable business models and stimulating innovativeness [54]. However, innovations in the initial period do not create value. On the contrary, they require investment, and often the use of dynamic capabilities to create new products and the flow of money are opposed to one another, and only in the long run do innovations turn into cash [118]. It turns out that the use of dynamic capabilities to create innovation also stimulates the development of technological and marketing skills, which can later be used for the development and commercialization of new products [55].

#### 2.3.4. Maintaining revenues at the current level as a value capture mechanism

Dynamic capabilities can mediate a firm’s valuable, rare, inimitable and non-substitutable resources to improve value capture and performance [119]. It turns out that environmental dynamism negatively influences the contribution of ordinary capabilities and positively influences the contribution of dynamic capabilities to a firm’s performance [17]. What is more, heterogeneity strengthens the contribution of dynamic capabilities to a firm’s performance.

### 2.4 Contextual factors

In terms of the characteristics used in research on dynamic capabilities, strategic goals and SMEs, it can be clearly seen that the most frequently used are the age [70,120–122] and size of a company [57,60,62]. It can even be said that they are genetic. Their impact has been repeatedly described in various contexts, *e*.*g*., in terms of innovation [123], company competitiveness [121] and SME performance [124].

### 2.5 Formulating hypotheses

**Fig 1:**
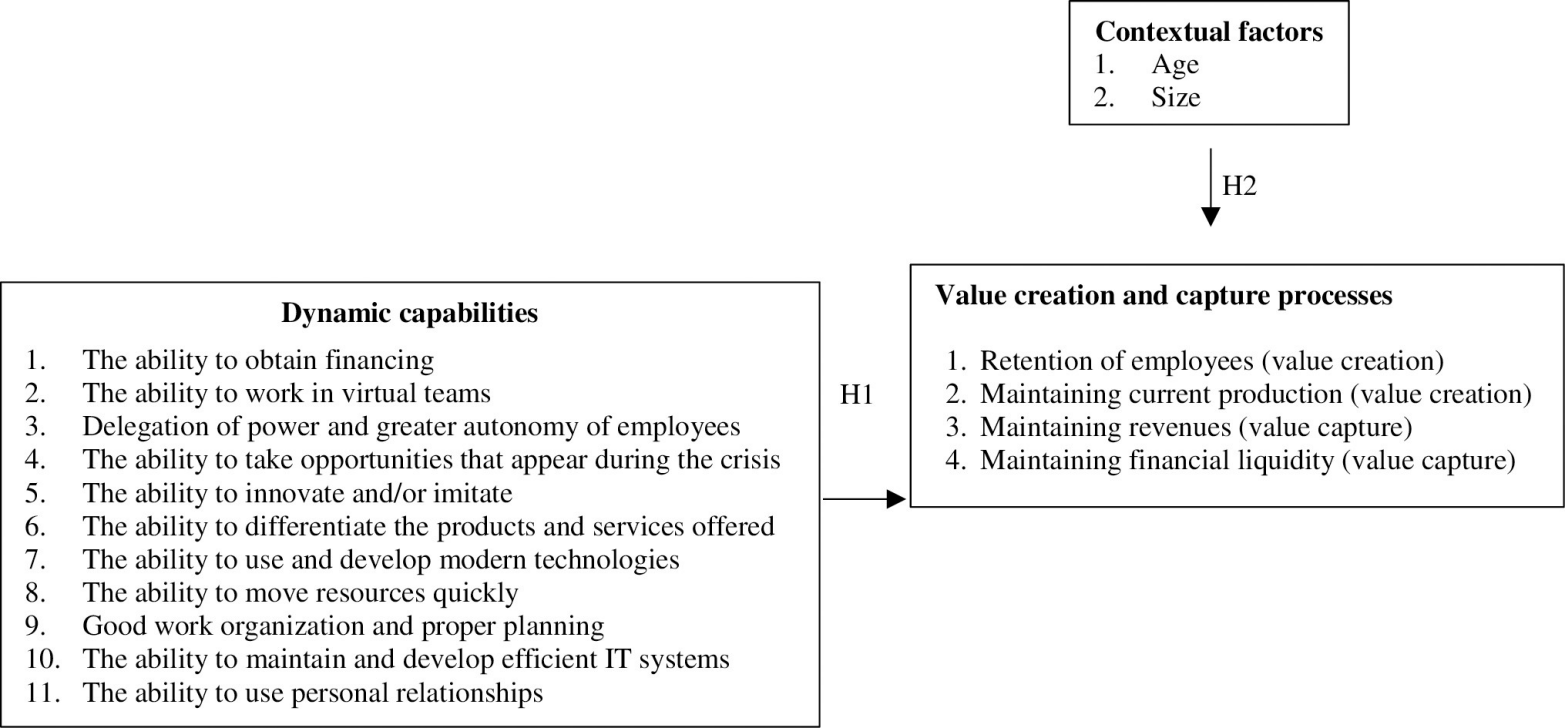
Dynamic capabilities influencing the value creation and capture processes.

The above analysis of research conducted so far in the field of dynamic capabilities—perceived in the context of the value creation and capture processes—led us to formulate the conceptual model presented below (Fig 1). Consequently, we propose the following two hypotheses:

*H1—Dynamic capabilities have influenced value creation and capture during the Covid-19 crisis*,
*H2—Contextual factors have influenced value creation and capture during the Covid-19 crisis*.

## 3. Empirical analysis

### 3.1. Data collection and sample

The data presented in this study come from empirical research carried out in April 2020. Its purpose was to identify and evaluate second order dynamic capabilities, understood as crisis responses and activities undertaken by Polish firms in the first period after the economic lockdown resulting from the coronavirus pandemic. The principal part of the research was conducted using the CAWI method among a random sample of 151 micro, small and medium-sized companies. The characteristics of the companies are presented in Table 2. As can be seen, the vast majority conducted service activities (90.7%), belonged to the medium-sized enterprises group (65.6%) and had been operating on the market for between 21 and 30 years (49%).

**Table 2:** The structure of the sample.

Characteristics	% in sample
Sector	
Production	5.3
Trade	7.3
Services	90.7
Firm size (no. of employees)	
< 9	8.6
10–49	25.8
50–249	65.6
Age of firm (years)	
< 10	17.2
11–20	20.5
21–30	49.0
31–40	4.0
> 41	9.3

### 3.2. Variables

Table 3 presents a description and scale for all the variables included in the model. Both the explained (dependent) and explanatory (independent) variables were measured using the seven-point Likert scale. In the case of the explained variables, respondents were asked to assess how important a specific value creation and capture outcome is for them, and in the case of the explanatory variables—how important a given dynamic capability is for surviving a crisis. As far as the company age, which acted as a control variable, was concerned, a logarithm was applied to the calculations. In turn, in terms of the number of employees, an ordinal variable was used with the following values: 1—micro, 2—small, 3 –medium-sized company.

**Table 3:** Summary of variables.

Description	Label	Type	Dimension
*Explained variable*			
Retention of existing employees	y_1_	Ordinal	SME outcomes
Maintaining financial liquidity	y_2_	Ordinal	SME outcomes
Maintaining revenues at the current level	y_3_	Ordinal	SME outcomes
Maintaining the current production level	y_4_	Ordinal	SME outcomes
*Explanatory variables*			
The ability to obtain financing	x_1_	Ordinal	Dynamic capabilities
The ability to work in virtual teams	x_2_	Ordinal	Dynamic capabilities
Delegation of power and greater employee autonomy	x_3_	Ordinal	Dynamic capabilities
The ability to exploit opportunities that arise during the crisis	x_4_	Ordinal	Dynamic capabilities
The ability to innovate and/or imitate	x_5_	Ordinal	Dynamic capabilities
The ability to diversify the products and services offered	x_6_	Ordinal	Dynamic capabilities
The ability to use and develop modern technologies	x_7_	Ordinal	Dynamic capabilities
The ability to move resources quickly	x_8_	Ordinal	Dynamic capabilities
Good work organization and proper planning	x_9_	Ordinal	Dynamic capabilities
The ability to maintain and develop efficient IT systems	x_10_	Ordinal	Dynamic capabilities
The ability to use personal relationships	x_11_	Ordinal	Dynamic capabilities
*Control variables*			
Age–number of years since business was founded	x_12_	Numerical	Contextual factors
Size–number of workers employed	x_13_	Ordinal	Contextual factors

### 3.3. Methods

In our research we examined a group of enterprises, however our research involved human participants, since we asked managers to answer survey questions. We informed participants that the survey is anonymous. We analyzed data anonymously, and we did not ask about any personal information. Therefore our research, in accordance with the recommendations of the National Science Center (https://ncn.gov.pl/sites/default/files/pliki/2016_zalecenia_Rady_NCN_dot_etyki_badan.pdf), which are the basis for drawing up guidelines for conducting research at our Universities (https://ue.poznan.pl/pl/badania-naukowe-uep,c458/komisja-ds-etyki-badan-naukowych,a60579.html; https://www.ue.katowice.pl/uczelnia/o-uczelni/kodeks-etyczny.html; https://www.wf.cm.umk.pl/panel/wp-content/uploads/1_UCHW-SENAT-2017-179-za%C5%82.pdf), did not require approval of ethics committees.

In order to find out how dynamic capabilities influence the processes of value creation and capture, we used ordered logistic regression, whose specification is an extension of the binary model specification to more thresholds. The following equation describes the model:

$$y^{*}=x^{\prime }\beta +u$$ (1)

where y* is the exact but unobserved dependent variable, *x’* is the vector of independent variables, *u* is the error term, and *β* is the vector of regression coefficients which we wish to estimate [125]. To estimate the model, we use the maximum likelihood estimation method and the STATA.16 software package.

### 3.4. Results

In the first step, Cronbach’s alpha test, the Kaiser-Meyer-Olkin test and the Barlett’s test were conducted. The results are shown in Table 4.

**Table 4:** Measurement properties.

Variable	Cronbach’s Alpha Test	Kaiser-Meyer-Olkin Test	Barlett’s Test
Explained	0.833	0.753	256.737*
Explanatory	0.875	0.852	761.055*

* *p* < 0.000.

According to the Cronbach’s alpha and Kaiser-Meyer-Olkin test results, the reliability of the research tool was confirmed. These values are acceptable for this type of analysis [126]. The authors are aware that some of the factors selected for the study are correlated with one another (Appendix 1). However, this is due to the fact that they relate to one phenomenon occurring in an organization.

The model estimation results are presented in Table 5.

**Table 5:** Ordered logistic regression for SME goals during the *coronavirus crisis*.

Variables	y1		y2		y3		y4	
	Coef.	S.E.	Coef.	S.E.	Coef.	S.E.	Coef.	S.E.
x_1_	0.147	0.091	0.649***	0.163	0.332***	0.097	0.225**	0.091
x_2_	-0.203	0.143	-0.800***	0.292	-0.202	0.151	-0.044	0.142
x_3_	0.258*	0.146	0.332	0.245	0.196	0.154	0.253*	0.149
x_4_	-0.283**	0.119	0.411*	0.218	-0.050	0.126	0.041	0.122
x_5_	0.058	0.138	0.565**	0.251	0.193	0.151	0.011	0.152
x_6_	-0.154	0.128	-0.447*	0.236	-0.221*	0.129	-0.246*	0.129
x_7_	0.019	0.130	-0.437*	0.234	0.091	0.141	-0.028	0.136
x_8_	-0.024	0.128	-0.294	0.233	-0.243*	0.138	-0.006	0.134
x_9_	0.241	0.154	0.679**	0.267	0.110	0.163	0.173	0.155
x_10_	0.184	0.148	-0.042	0.254	0.233	0.151	0.330**	0.150
x_11_	0.042	0.108	0.116	0.187	0.139	0.114	0.163	0.113
x_12_	0.829	0.637	-0.208	1.150	0.180	0.679	0.726	0.629
x_13_	0.085	0.296	0.658	0.481	0.559*	0.294	0.742***	0.289
_cut1	-0.838	1.256	0.181	2.139	1.137	1.253	3.901	1.228
_cut2	-0.030	1.224	0.619	2.107	1.550	1.243	4.698	1.227
_cut3	0.472	1.220	1.277	2.084	2.201	1.250	5.009	1.231
_cut4	1.335	1.221	1.966	2.079	2.828	1.263	5.790	1.249
_cut5	2.270	1.228	3.567	2.107	3.851	1.283	6.787	1.277
_cut6	2.987	1.236			4.701	1.298	7.105	1.307
Log-likelihood	-180.20		-63.134		-162.809		-175.768	
LR chi^2^ (13)	38.10		56.98		46.26		74.80	
Prob > chi^2^	0.0003		0.0000		0.0000		0.0000	
Pseudo R^2^	0.0956		0.3109		0.1244		0.1754	

* **p-Value ≤ 0.1**.

** **p-Value ≤ 0.05**.

*** **p-Value ≤ 0.01.**

As can be seen, all the models are statistically significant. The test statistic for the likelihood-ratio test, used to verify the null hypothesis that a model with only *k* thresholds (_cut1 - _cut6) is as good as the estimated model, is 38.10, 56.98, 46.26 and 74.80 respectively. At 13 degrees of freedom, the empirical significance level for these statistics is practically 0, so we reject the null hypothesis in favor of the alternative hypothesis that the estimated models are better than those that only account for thresholds. It is also worth emphasizing that in the case of the liquidity maintenance model (y_2_), the explanatory variables provide a relatively large amount of information (psudo-R^2^ = 0.3109). The odds ratios for the regression coefficients for all the models are presented in Appendix B.

When businesses are striving to retain their current employees (y_1_), two alternatives turned out to be statistically significant—delegation of power and greater autonomy of employees, and the ability to take advantage of opportunities that emerge during a crisis.

The most statistically significant changes occurred in the case of SMEs striving to maintain current liquidity (y_2_). These capabilities were the ability to obtain financing, the ability to work in virtual teams, the ability to use opportunities that arise during the crisis, the ability to innovate and/or imitate, the ability to differentiate between products and services offered so far, the ability to use and develop modern technologies, and good organization of work.

On the other hand, in the case of the model describing the efforts of SMEs to maintain revenues at the current level (y_3_), the following variables turned out to be significant: the ability to obtain financing, the ability to diversify the products and services offered so far, the ability to move resources quickly, and the size of the company.

In the model describing the efforts of organizations to maintain their production level and market share (y_4_), the following five variables are important: the ability to obtain financing, delegation of power and greater autonomy of employees, the ability to differentiate between products and services offered so far, the ability to maintain and develop efficient IT systems, and the size of the company.

Below, we present our interpretation of the results obtained. When analyzing the results, it is worth looking at the odds ratios, however, it should be remembered that the interpretations of the odds ratios are important in a given set of explanatory variables of the model and assuming *ceteris paribus*. This means that each time we are talking about two organizations with identical values for the explanatory variables, except for the one we are explaining. The conducted research procedure showed the existence of positive dependencies between the ability to obtain financing and maintaining financial liquidity (firms for which obtaining financing is essential to survive in a crisis, *ceteris paribus*, have a 91% greater chance of maintaining financial liquidity than firms for which this ability is less important), and between maintaining revenues at the current level (firms for which obtaining financing is vital for surviving in a crisis, *ceteris paribus*, have a 39% greater chance of maintaining revenues at the current level than firms for which this ability is less important) and maintaining current production (firms for which obtaining financing is critical for surviving in a crisis, *ceteris paribus*, have a 25% greater chance of keeping current production than firms for which this ability is less important).

The identified dependency between the ability to obtain financing and maintaining financial liquidity seems to be notable (ignoring, of course, the issue of the impact on short-term goals of dynamic abilities, which have the character of competencies developed in the long term). However, the identified positive dependency between the ability to obtain financing and maintaining revenues at the current level and maintaining the current production level requires a comment. On the one hand, the identified phenomena suggest that the studied companies were able to react very quickly to signals from the environment, which was most likely possible, as Zhang [84] points out, thanks to efficient IT systems. On the other hand, however, the identified dependency indicates the ability of the surveyed companies to determine the necessary financial resources necessary to secure the implementation of activities that allow sales revenues to be maintained, as well as maintain or rebuild production capacity and secure supply chains at risk during the crisis [114], which, in the long term, will determine their competitive position and will allow, in the event of a quick rebound in demand, for them to increase their market share.

The identified negative dependency between the ability to work in virtual teams and maintaining financial liquidity may indicate problems with cost management arising in the case of the physical separation of employees (firms for which the ability to work in virtual teams is important for surviving in a crisis, *ceteris paribus*, have a 55% less chance of a lower, not higher, assessment of value capture by maintaining financial liquidity than firms for which this ability is less important). However, problems with maintaining financial liquidity are probably a consequence of a reduction in sales revenues, which is the main driver of value creation and capture.

The positive dependency between the ability to delegate power and greater autonomy of employees and the retention of existing employees points to the importance of self-organization and, as a consequence of decentralization and autonomy, the ability to seek new actions in times of crisis conditions (SMEs, for which delegation of power and greater employee autonomy are essential for surviving in a crisis, *ceteris paribus*, have a 29% greater chance of a higher, not lower, assessment of the importance of value creation by retaining existing employees than SMEs for which this ability is less important). Companies in which employees have high decision-making autonomy, are able to react quickly to changes in the environment, which affects process innovations [47] and maintains the level of sales, thanks to which the need for actions related to employee recruitment is limited.

The identified negative dependency between the ability to take advantage of opportunities emerging during the crisis and the retention of the existing employees indicates that the surveyed companies abandon some of their activities and remodel their business models to identify and exploit opportunities in the environment, which makes it necessary to retain human resources that do not fit the new challenges (SMEs for which exploiting opportunities during a crisis is essential to creating value, *ceteris paribus*, have a 26% greater chance of a higher, rather than a lower assessment of the importance of value creation by retaining existing employees than SMEs for which this ability is less important).

The research procedure showed a positive dependency between the ability to take advantage of opportunities emerging during a crisis and maintaining financial liquidity, which indicates the ability of companies to sense changes in the environment and to take advantage of opportunities by reconfiguring their resources and competences, which is also indicated by Harreld, O’Reilly, Tushman [53] (SMEs for which the use of opportunities arising during a crisis is important for surviving the crisis, *ceteris paribus*, have a 51% greater chance of a higher, not lower, assessment of the importance of value capture by maintaining financial liquidity than SMEs for which this ability is less important).

In the case of the dynamic capability relating to innovating and imitating, the researched companies that innovate or imitate have a 76% greater chance of capturing value by maintaining financial liquidity. In turn, the ability to diversify the products and services offered so far reduces the chances of capturing value through maintaining financial liquidity by 36%, maintaining revenues at the current level by 20%, and creating value by maintaining the current production level by 22%. In contrast, the dynamic capability based on using and developing modern technologies reduces the chances of maintaining financial liquidity by 35% and the ability to move resources quickly reduces by 22% the value capture by maintaining revenues at the current level.

In the case of the dynamic capability relating to good work organization and proper planning, an influence was observed relating to value capture through maintaining financial liquidity. It can be noticed that firms in which good work organization and proper planning are more important for surviving in a crisis, *ceteris paribus*, have a 97% greater chance of maintaining financial liquidity than firms for which this capability is less important.

A similar observation applies to the ability to maintain and develop efficient IT systems. Here, a dependency was observed in relation to value creation through maintaining current production. Firms for which the ability to maintain and develop efficient IT systems is more critical for survival in a crisis, *ceteris paribus*, have a 39% greater chance of maintaining current production than SMEs for which this ability is less important.

However, in the case of company size (a control variable), it can be stated that larger companies have a 74% greater chance of maintaining revenues at the current level, and a 110% greater chance of maintaining the current production level than smaller companies.

## 4. Discussion

An important task for contemporary strategic management is to look for sources of value, and then create and capturing this value. This task has been identified from various theoretical perspectives. In this paper we have posited that one of the lenses through which the value creation and capture processes should be looked at is the dynamic capability perspective. The question of responding quickly and adequately to unexpected events in the environment, while at the same time creating and capturing value, seems to have become even more significant during the turbulent times that have resulted from the pandemic.

Dynamic capabilities, which influence the organization-environment fit, facilitate a firm’s survival during times of crisis. The objective of this paper was to identify the most common indicators of a firm’s dynamic capabilities and to relate them to the processes of value creation and value capture. The most significant conclusions are as follows: (a) there is a positive relation between the ability to exploit opportunities emerging during a crisis and maintaining financial liquidity, (b) imitating and innovating increases the chances of maintaining financial liquidity, (c) diversifying products and services decreases financial liquidity, (d) developing modern technologies reduces the chances of maintaining financial liquidity, (e) orchestrating resources reduces the chances of maintaining revenues at the current level.

We have also found that firms that plan strategically have a greater chance of maintaining financial liquidity. Firms that develop efficient IT systems have a greater chance of maintaining current production. When the control variables are applied, large companies have a greater chance of maintaining revenues at the current level and maintaining the current production level.

The dynamic capability perspective focuses attention on skillful modification of a firm’s strategic potential in order to reach above-average performance [3]. Dynamic capabilities can also be connected with certain potential-modifying processes and mechanisms such as product innovation, network building and purchases or investments [127]. Understanding the nature of dynamic capabilities makes it possible to explore more deeply the difference between ordinary and dynamic capabilities: operational capabilities (the first level) include routine activities, while dynamic capabilities encompass second order and higher order capabilities (the second level) [10]. It has been noted that strong dynamic capabilities are a sound basis for flexibility and allow for uncertainty to be dealt with strategically [11]. Therefore, we found it appropriate to analyze dynamic capabilities during the Covid-19 pandemic. Following the suggestion of Teece [6] that first-order dynamic capabilities include managerial decisions, responses and activities during uncertainty, in this paper we have concentrated on identifying first-order dynamic capabilities during the coronavirus crisis that resulted from the first economic lockdown. Scholars researching dynamic capabilities associate the construct mainly with seeking new opportunities, acquiring new resources or creating value [103], therefore we assumed that value creation and capture will act as a dependent variable in our study. This falls under the vein of thinking that dynamic capabilities concentrate on goal-oriented activities used to create and capture value. Research to date has distinguished various substantial capabilities and overall dynamic capabilities such as product innovation, network building, purchases or investments (Eisenhardt, Martin, 2000) [127], keeping good relations with customers, human resource management, maintaining high product quality, building a proper organizational structure (Karna et al., 2016) [35], identifying opportunities for creating and capturing new value, mobilizing strategic potential in pursuit of opportunities, and transforming the company and its resource base for the future (Teece, 2007) [5]. In most cases, the dynamic capabilities identified in this research fall into the theoretical categories.

Research shows that dynamic capabilities, seen as a company’s ability to adapt to a turbulent environment, are crucial during times of crisis. Lack of preparedness [31] and a low level of innovativeness [32] force many companies to go under. The dynamic capabilities identified through our research confirm the necessity during crises of developing such dynamic capabilities as the ability to obtain financing, the ability to innovate, to move resources quickly, to seize new opportunities, as well as to build relations with stakeholders [46] and develop IT systems and proper organizational structures [7].

Some research provides evidence on the relationship between dynamic capabilities and performance [35]. Our research has demonstrated that the value creation and value capture processes that translate into overall performance are largely shaped by a firm’s dynamic capabilities during times of crisis.

The contribution of this paper is three-fold. First, it adds to the theoretical literature on dynamic capabilities by demonstrating that it is important to delineate between first-order dynamic capabilities and overall dynamic capabilities. While the latter should be sought after by strategic managers as they shape a company’s future, it is not possible to study higher dynamic capabilities without knowing which first-order dynamic capabilities constitute them.

Second, this paper demonstrates that certain dynamic capabilities shape the value creation and value capture processes. In particular, we found that the ability to exploit opportunities during a crisis, imitating and innovating, as well as developing modern technologies all impact maintaining financial liquidity. On the other hand, resource orchestration negatively influences revenue retention, which suggests that it is a capital-intensive process.

Third, with relatively little empirical research carried out into crisis responses in Central and Eastern Europe during the Covid-19 pandemic, it can be expected that the lengthening crisis and its economic impact will produce substantially more research in this area. However, this paper primarily contributes to dynamic capability management studies by identifying the most important first-order dynamic capabilities–managerial responses during uncertainty–and their influence on value-related aspects of a firm’s performance.

Several categories of practical implications are crucial with regard to a company’s actions taken in order to survive a crisis. The first category is to maintain financial liquidity and activity. To ensure this, managers should create: a) the ability to obtain financing (i.e. credit lines, cash reserves etc.), and thanks to sources of finance the ability to retain revenues (by financing marketing and sales processes) and production levels (by financing materials and others resources for production); b) the ability to take advantage of opportunities emerging during the crisis (i.e. to finance new activities); c) the ability to make use of innovations and imitations (which could optimize costs); and d) good work organization and proper planning (i.e. control of processes, limiting fixed costs and ensuring production fluency). In the same category, in order to maintain financial liquidity, managers should avoid: a) diversification of products (because in times of crisis all resources should be moved to the core product due to costs); and b) modern technologies (high levels of technology are costly and can be unstable). This category (maintaining financial liquidity) is the first and most important in ensuring the survival of a company, not only in times of crisis. The second category is retaining employees and activity. To ensure this, managers should: a) create virtual teams; and b) delegate power and foster greater autonomy of employees. Giving greater autonomy to employees also results in the fluidity of processes. The third category is to maintain production levels by taking care of IT systems responsible for production management (i.e. automation etc.)

Creating virtual teams can also have negative implications for financial liquidity, when control of costs and work efficiency is lost. Taking advantage of opportunities that emerge during a crisis has negative implications for retaining existing employees, most probably because new employees with different skills need to be sought due to new activity, new products or even a new business model.

We are aware that this study has some limitations. Primarily, as we intended to quickly capture the first organizational reactions after the March 2020 economic lockdown, we targeted as many respondents as were willing to take part in the survey. The sample, being a random selection of the first organizations that responded, presents one of the limitations–the structure of the sample shows overrepresentation of service companies. The researched organizations were also of various size and age, which also creates possibilities for varying interpretation of the research results.

The survey design and selection of measurements also raises some possible limitations. We are aware that the list of an organization’s first-order capabilities, which reflect their dynamic capabilities during times of crisis, is not exhaustive or complete. However, these are the capabilities that the respondents assessed as important. The value creation indicators (maintaining the workforce and production, and securing current markets) and the value capture indicators (cash flow and revenue stream) that we have chosen do not complete the full scope of the theoretical framework found in the literature.

The research context creates yet another limitation. Although Poland represents a rich context for studying dynamic capabilities as being a country with opportunity-based entrepreneurship, dynamic GDP growth and low unemployment even during the coronavirus crisis, it is not a representative country for the region. Therefore, the conclusions from this study will not necessarily be a basis for overall generalization.

Future research would therefore need to address these issues identified above. Firstly, a more longitudinal approach to the research could be adopted. We are currently analyzing results from the second lockdown in October 2020, as well as researching the current (March 2021) lockdown. It would be interesting to compare the data and see how the structure and composition of dynamic capabilities have changed in the organizations studied. The possible slow-down of the pandemic, the acceleration of the vaccination program and herd immunity will lead companies towards a more stable environment. It would also be possible to carry out similar research to examine the structure and composition of dynamic capabilities after the coronavirus crisis. For future research, a revised version of the questionnaire could be used, and more precise measures of dynamic capabilities as well as value creation and capture could be implemented. It would also be recommended to carry out a comparative analysis to identify the structure of dynamic capabilities in other CEE countries, or even countries on other continents, in order to identify common dynamic capabilities developed during the coronavirus crisis worldwide.

Overall, this study represents an attempt to develop new knowledge and understanding of dynamic capability development during an unexpected crisis, and the impact of such capabilities on the value creation and value capture processes in companies. Assuming that a similar situation could occur in future pandemics, this paper may help guide future research in this regard.

## Appendix A. Kendall Tau-b correlation matrix

**Table:** 

Variables	y_1_	y_2_	y_3_	y_4_	x_1_	x_2_	x_3_	x_4_	x_5_
y_1_	1.000								
y_2_	.401**	1.000							
y_3_	.381**	.478**	1.000						
y_4_	.401**	.443**	.497**	1.000					
x_1_	.170*	.341**	.307**	.292**	1.000				
x_2_	-0.003	-0.040	0.054	.212**	.145*	1.000			
x_3_	0.084	0.079	0.114	.246**	.150*	.611**	1.000		
x_4_	-.160*	.160*	0.028	0.101	.142*	.315**	.299**	1.000	
x_5_	-0.038	.147*	0.078	.135*	0.103	.338**	.356**	.525**	1.000
x_6_	-.187**	-0.002	-0.103	-0.065	0.095	.249**	.251**	.481**	.501**
x_7_	0.048	0.085	.155*	.231**	.242**	.366**	.285**	.397**	.502**
x_8_	-0.013	0.036	0.008	.149*	.191**	.382**	.378**	.396**	.498**
x_9_	.205**	.274**	.294**	.397**	.248**	.293**	.319**	.224**	.322**
x_10_	.193**	.192**	.298**	.417**	.253**	.381**	.333**	.262**	.342**
x_11_	0.016	0.103	0.111	.154*	0.115	.212**	.199**	.371**	.291**
x_12_	.126*	0.042	0.052	0.108	0.011	-0.034	-0.044	-.170**	-0.106
x_13_	.166*	0.141	.217**	.281**	0.018	-0.006	0.042	-0.125	-0.100
Variables	x_6_	x_7_	x_8_	x_9_	x_10_	x_11_	x_12_	x_13_	
y_1_									
y_2_									
y_3_									
y_4_									
x_1_									
x_2_									
x_3_									
x_4_									
x_5_									
x_6_	1.000								
x_7_	.306**	1.000							
x_8_	.449**	.457**	1.000						
x_9_	.146*	.419**	.300**	1.000					
x_10_	0.103	.456**	.294**	.627**	1.000				
x_11_	.260**	.341**	.219**	.320**	.254**	1.000			
x_12_	-0.110	-0.096	-0.011	-0.021	-0.097	-0.097	1.000		
x_13_	-.188**	0.030	-0.108	0.130	.143*	-.141*	.217**	1.000	

** Significant correlation at the level of 0.01.

* Significant correlation at the level of 0.05.

## Appendix B. Odds ratio for y_1_, y_2_, y_3_, y_4_ models

**Table:** 

Variables	y_1_	y_2_	y_3_	y_4_
x_1_	1.158	1.913	1.394	1.253
x_2_	0.815	0.449	0.816	0.956
x_3_	1.295	1.394	1.217	1.288
x_4_	0.735	1.509	0.950	1.042
x_5_	1.060	1.759	1.213	1.011
x_6_	0.856	0.639	0.801	0.781
x_7_	1.019	0.645	1.096	1.028
x_8_	0.976	0.744	0.783	0.993
x_9_	1.273	1.972	1.117	1.189
x_10_	1.202	0.957	1.263	1.392
x_11_	1.043	1.123	1.149	1.178
x_12_	2.291	0.811	1.198	2.068
x_13_	1.089	1.931	1.746	2.102
